# Recent Developments of Flexible and Stretchable Electrochemical Biosensors

**DOI:** 10.3390/mi11030243

**Published:** 2020-02-26

**Authors:** Xudong Yang, Huanyu Cheng

**Affiliations:** 1 Key Laboratory of Optoelectronic Technology & Systems (Ministry of Education), Chongqing University, Chongqing 400044, China; xudong_yang@outlook.com; 2 Department of Automotive Engineering, Beihang University, Beijing 100191, China; 3 Department of Engineering Science and Mechanics, The Pennsylvania State University, University Park, PA 16802, USA; 4 State Key Laboratory of Digital Manufacturing Equipment and Technology, Huazhong University of Science and Technology, Wuhan 430074, China

**Keywords:** electrochemical biosensors, wearable devices, flexible and stretchable, template and non-template printing methods, health monitoring

## Abstract

The skyrocketing popularity of health monitoring has spurred increasing interest in wearable electrochemical biosensors. Compared with the traditionally rigid and bulky electrochemical biosensors, flexible and stretchable devices render a unique capability to conform to the complex, hierarchically textured surfaces of the human body. With a recognition element (e.g., enzymes, antibodies, nucleic acids, ions) to selectively react with the target analyte, wearable electrochemical biosensors can convert the types and concentrations of chemical changes in the body into electrical signals for easy readout. Initial exploration of wearable electrochemical biosensors integrates electrodes on textile and flexible thin-film substrate materials. A stretchable property is needed for the thin-film device to form an intimate contact with the textured skin surface and to deform with various natural skin motions. Thus, stretchable materials and structures have been exploited to ensure the effective function of a wearable electrochemical biosensor. In this mini-review, we summarize the recent development of flexible and stretchable electrochemical biosensors, including their principles, representative application scenarios (e.g., saliva, tear, sweat, and interstitial fluid), and materials and structures. While great strides have been made in the wearable electrochemical biosensors, challenges still exist, which represents a small fraction of opportunities for the future development of this burgeoning field.

## 1. Introduction

As personal healthcare starts to gain skyrocketing popularity, various wearable sensors have been developed for the health monitoring of the individual [1,2,3,4]. With relatively simple design, physical sensors have been explored to capture physical (e.g., temperature [5,6], motion [7,8], respiration rate [9,10], and gas exposure [11,12] among others) and electrophysiological signals (e.g., electrocardiogram, ECG [13]; electromyogram, EMG [14]; and electroencephalogram, EEG [15]; among others). However, it is still highly desirable to capture chemical information for reflecting complete physiological conditions from the children to the elderly. With a recognition element (e.g., enzymes, antibodies, nucleic acids, ions) to selectively react with the target analyte [16,17], electrochemical biosensors can convert the types and concentrations of biochemical changes in the body into electrical signals [18,19]. However, traditional electrochemical biosensors are rigid and bulky. Because of the mismatch in material and geometry, their applications are limited on the soft, hierarchically textured surfaces of the human body [20,21]. Therefore, the development of flexible and stretchable electrochemical biosensors becomes attractive [22,23,24,25,26,27,28]. 

**Figure 1:**
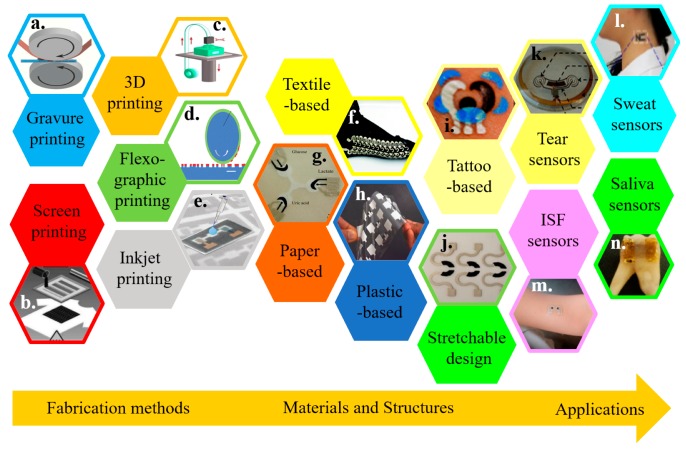
Overview of wearable electrochemical biosensors. Representative fabrication approaches include (**a**) gravure printing, reproduced with permission from [49], Copyright 2015, Springer; (**b**) screen printing, reproduced with permission from [50], Copyright 2013, Wiley-VCH, (**c**) 3D printing, reproduced with permission from [51], Copyright 2016, Wiley-VCH, (**d**) flexographic printing, reproduced with permission from [52], Copyright 2010, The Royal Society of Chemistry, and (**e**) inkjet printing, reproduced with permission from [53]. Copyright 2017, American Chemical Society. Wearable electrochemical sensors can be integrated on various substrate materials, including (**f**) fabric/textile, reproduced with permission from [54], Copyright 2014, Wiley-VCH; (**g**) paper, reproduced with permission from [55], Copyright 2014, Wiley-VCH; (**h**) plastic thin films, reproduced with permission from [56], Copyright 2012, Macmillan Publishers Limited; (**i**) temporary tattoo substrates, reproduced with permission from [57], Copyright 2012, The Royal Society of Chemistry; along with (**j**) stretchable structures, reproduced with permission from [58], Copyright 2015, American Chemical Society. Applying the resulting wearable electrochemical sensors can analyze a variety of body fluids, including (**k**) tear, reproduced with permission from [59], Copyright 2015, IOP Publishing Ltd.; (**l**) sweat, reproduced with permission from [60], Copyright 2013, American Chemical Society; (**m**) interstitial fluid (ISF), reproduced with permission from [61], Copyright 2014, American Chemical Society, and (**n**) saliva, reproduced with permission from [62], Copyright 2012, Macmillan Publishers Limited.

The recent development in cost-effective fabrication approaches includes various additive manufacturing or printing technologies. Integrating electrochemical biosensors on soft substrates with these approaches renders flexible and stretchable properties for wearable applications (Figure 1) [2,29,30,31,32,33,34]. Printing electrochemical sensing materials directly onto soft textiles of daily clothes allows for timely monitoring of critical information, without compromising the level of comfort or function of the garment [35,36,37]. However, textile-based biosensors are limited to certain regions because of the need for pliably conformal or intimate contact with biofluids for high-fidelity detection. Additionally, not all types of textiles are suitable to integrate sensing materials [38,39,40]. As an alternative, other flexible thin-film substrates (e.g., paper and plastic) have been explored [41,42,43,44,45,46]. However, the intrinsic fracture limit of the flexible thin-film materials is small (e.g., <1% for paper). Thus, the deformation of flexible electrochemical biosensors is limited. Considering a stretchable property is needed to form an intimate contact with the textured skin surface and to deform it with various natural skin motions, stretchable materials and structures have been exploited to ensure the effective function of an electrochemical biosensor (Figure 1) [32,47,48].

In this mini-review, we summarize the latest development of wearable electrochemical biosensors. Their working principles, target biofluids, fabrication approaches, and wearable electrochemical biosensors based on various stretchable materials and structures are reviewed. Following the introduction of the working principles of different electrochemical biosensors in Section 2, we discuss the variety of target biofluids (e.g., sweat, tear, saliva, and interstitial fluids) for wearable electrochemical biosensors to sample and analyze in Section 3. Next, Section 4 focuses on a few representative cost-effective fabrication approaches such as additive manufacturing or printing to integrate electrochemical biosensors on various flexible and stretchable substrates, followed by a conclusion and future perspective in Section 5.

## 2. Principles of Electrochemical Biosensors

**Figure 2:**
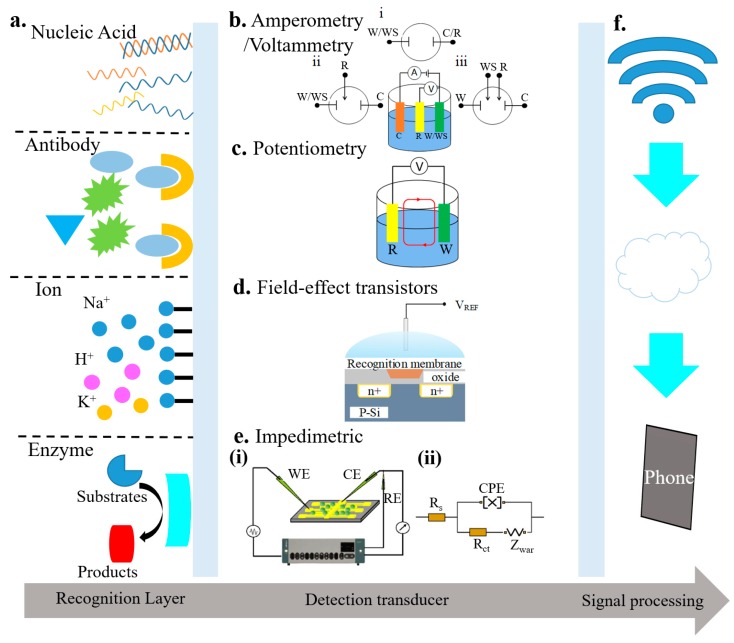
Schematic of electrochemical biosensors that include (**a**) a recognition element (e.g., nucleic acid, antibody, ions, or enzyme) and a detection transducer, along with a corresponding signal processing unit; reproduced with permission from [63]; Copyright 2016, The Royal Society of Chemistry. The detection transducer could be (**b**) amperometric/voltammetric sensors with (i) two-, (ii) three-, or (iii) four-electrode configuration (reproduced with permission from [63]; Copyright 2016, The Royal Society of Chemistry), (**c**) a potentiometric sensor (reproduced with permission from [64]; Copyright 2014, National Academy of Sciences), (**d**) a field-effect transistor sensor (reproduced with permission from [65]; Copyright 2017, Elsevier), or (**e**) an impedimetric sensor (reproduced with permission from [66], Copyright 2008, Elsevier.) (**f**) Signal processing element that may include wireless communication module and processing units such as phones (reproduced with permission from [65]; Copyright 2017, Elsevier).

Electrochemical biosensors often consist of a recognition element and a sensor element (Figure 2). The recognition element could be the nucleic acid, antibody, ions, or enzyme. The sensing element may rely on amperometry/voltammetry, potentiometry, field-effect transistors, or impedimetry [63,64,65,66]. These different types of electrochemical biosensors are also compared in Table 1 [64,65,66,67]. The transduced signals from the sensing element can be transmitted through a wired or wireless communication module. The analysis of the data can then help inform the health condition.

### 2.1. Amperometric/Voltammetric Biosensors

By probing the potential–current relationship, the amperometric or voltammetric biosensors can detect electroactive species present in biological samples. In voltammetric biosensors, the applied potential is varied to operate in either a linear or cyclic voltammetric mode. The target analyte can be identified by the peak potential, whereas its concentration could be informed by the peak current. By contrast with voltammetric biosensors, amperometric sensors relate the measured current with the concentration of a specific analyte at a fixed potential. Other than the Faradaic current from the reaction of the analyte, other sources of current also contribute to the measured current (collectively referred to as the background current). The background current includes the electrolysis of impurities, the electrolyte, and the electrode material, along with the capacitive current from the electrode/solution interface. Operating the electrode at a fixed potential can help eliminate the capacitive current. While the background current might be subtracted from the total current in some cases [68,69,70], it can be challenging in other cases as the background current may interact with the signal current in a non-linear manner. The electrochemical system in a simple amperometric/voltammetric cell configuration could consist of a few from two to four electrodes, i.e., a working, working sensing, counter, and reference electrodes (Figure 2a). In the amperometric biosensor, gold, carbon, or platinum represents the common choice for the working electrode. These electrode materials can provide good electron transfer towards the substrate in the reaction and maintain high activation energy for electron transfer in the competing reactions. Ag/AgCl often serves as the reference electrode to provide a fixed potential against which the potential of the working electrode is controlled and measured. The two-electrode configuration is simple. However, it has limited control of the potential on the surface of the working electrode with large currents, leading to a smaller linear range. To overcome this limitation, the counter electrode is employed to provide a more stable potential reference in the three-electrode configuration. In a four-electrode biosensor, redox recognition elements are immobilized on both the working and working sensing electrodes. The proximity of two working electrodes enhances redox recycling that helps regenerate electroactive species after their oxidation or reduction. Thus, this configuration is ideally suited for micro-scale interdigitated electrode arrays. Because redox recycling is only available with the redox bio-recognition element, four-electrode systems are not as commonly used as their three-electrode counterparts. Amperometric/voltammetric biosensors with different configurations are commonly used as immunosensors, enzyme biosensors, and pesticide monitors [71,72,73,74].

### 2.2. Potentiometric Biosensors

In potentiometric biosensors, an analyte recognition event is converted into a potential signal for sensing (Figure 2b). Local equilibrium is established across the recognition membrane (e.g., ion-selective members), leading to a change in the membrane potential. The information of the analyte is obtained from the potential difference between the working and reference electrodes. The most common potentiometric biosensors are capable of detecting pH, ions (e.g., F^−^, I^−^, CN^−^, Na^+^, K^+^, Ca^2+^, or NH^4+^), and gas (e.g., CO_2_ or NH_3_). The potential differences between the working and reference electrodes are proportional to the logarithm of the ion activity or gas fugacity (i.e., effective partial pressure or concentration) [71,72,73,74]. Although potentiometric biosensors are simple, of low cost, and highly selective, they have low sensitivity that limits their use in many applications [72]. In addition to the contribution from the sensor response, the current response in potentiometric biosensors also comes from the electrode double layer charging current that can be estimated by a double-layer capacitance model. The charging current is often considerable and difficult to remove or filter out, thereby limiting the resolution of potentiometric sensors [73].

### 2.3. Field-Effect Transistor (FET) Biosensors

In the ion-sensitive field-effect transistors (ISFETs), an ion-selective membrane is applied directly to the insulated gate of the field-effect transistors (Figure 2c). The ISFETs can also be used to determine the corresponding ion concentrations. When such ISFETs are coupled with a biocatalytic or biocomplexity layer, they become biosensors and are usually called either enzyme or immunological field-effect transistors (ENFETs or IMFETs). Unlike potentiometric and amperometric biosensors that use an electrode transducer, the ISFETs’ transducer is the gate oxide layer. The response of the ISFETs allows the output to be either the gate voltage or source-drain current by fixing one and measuring the other. The ISFET biosensors have been developed for enzyme sensing, antigen–antibody binding reaction measurements, and DNA detection [71,73,75].

### 2.4. Impedimetric Biosensors

Impedimetric biosensors measure the change in electrical impedance. This change often results from changes in capacitance and/or resistance of bio-interface characteristics for bio-recognition events. As a small sinusoidal stimulus voltage (or current) is imposed in a range of frequencies, the resulting current (or voltage) is measured in the impedimetric biosensor. Thus, it can inform bio-recognition events from the obtained phase and/or amplitude changes. Compared to potentiometric and amperometric biosensors, an important advantage of impedimetric biosensors is that they do not damage or disturb most bio-recognition events because of the applied stimulus sinusoidal voltage is negligibly small (usually 5–10 mV in amplitude) [74].

## 3. Target Biofluids for the Wearable Electrochemical Biosensors

**Figure 3:**
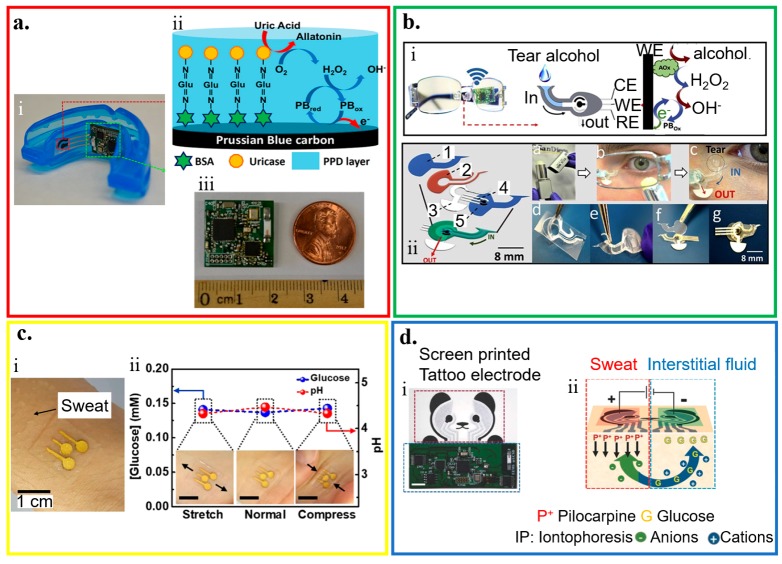
Application of wearable electrochemical biosensor for biofluid analysis. (**a**) Saliva analysis from (i) a mouthguard biosensor integrated with a wireless amperometric circuit board. (ii) Schematic of reagent layer of the chemically modified, printed Prussian Blue (PB) carbon working electrode containing uricase for the salivary uric acid (SUA) biosensor and (iii) optical image of the wireless amperometric circuit board; reproduced with permission from [76], Copyright 2015, Elsevier. (**b**) Tear analysis by (i) an eyeglasses platform consisting of wireless electronics and a fluidic device with (ii) its exploded view that shows (1) top polycarbonate membrane, (2) double-adhesive spacer, (3) paper outlet, (4) electrochemical (bio)sensor, and (5) bottom polycarbonate membrane; reproduced with permission from [93], Copyright 2019, Elsevier. (**c**) Sweat analysis from (i) an electrochemical biosensor attached to the skin wet with sweat, along with (ii) its performance under mechanical deformation; reproduced with permission from [85], Copyright 2018, American Chemical Society. (**d**) Interstitial fluid (ISF) analysis that relies on (i) a screen-printed glucose biosensor and a wireless flexible printed circuit board, with schematic illustrations of (ii) iontophoretic operation and layout of glucose biosensor; reproduced with permission from [92], Copyright 2018, Wiley-VCH.

Wearable electrochemical biosensors have been explored for human health monitoring through the analysis of saliva, tear, sweat, and interstitial fluid (Figure 3). Table 2 compares the representative electrochemical biosensors for these different target biofluids within the last five years.

### 3.1. Saliva Analysis

Human saliva is a watery substance that contains 99.5% water with electrolytes, mucus, white blood cells, epithelial cells, glycoproteins, enzymes, among others [94]. By leveraging the developments of biosensors, glucose and lactate in saliva can be non-invasively monitored by a cavitas sensor from the oral cavity. A wearable biosensor on a mouthguard is developed to monitor salivary lactate [77]. The fabrication starts with the printing of Ag/AgCl as the reference electrode and contacts (for interfacing the electrochemical analyzer) on a flexible PET substrate. Next, the Prussian Blue (PB)-graphite ink and LOx are coated on the working electrode (without LOx for the counter electrode), followed by a coating of an insulation layer. The resulting lactate sensor demonstrates high sensitivity of 0.553 μA·mM^−1^ and a low limit of detection 0.050 mM. Building on this work, a mouthguard electrochemical biosensor using the enzyme (uricase)-modified electrode from screen-printing along with an integrated wireless amperometric circuitry detects salivary uric acid with high sensitivity of 2.32 µA/mM (Figure 3a) [76]. 

By using non-toxic serine amino acid as linker molecules for the functionalization of nZrO_2_, a label-free and non-invasive biosensing platform can efficiently detect microRNA, an oral cancer biomarker [63]. This sensor has a low limit of detection of 0.01 ng/mL that is sufficient for the lower secretion level of targets in human saliva. And the biosensor also exhibits a linear detection range 0.01–29 ng/mL with a sensitivity of 0.295 μA mL/ng, along with a response time of 6 min and long-term stability up to 45 days. While the monitoring of saliva is of high interest for healthcare, significant challenges still exist for the saliva electrochemical biosensors. The complicated mixture in the saliva requires the sensor to be highly selective. The sensor also needs to maintain stable performance in such a high moisture environment. Additionally, the devices should be fully biocompatible due to the use in the mouth.

### 3.2. Tear Analysis

Electrolytes, metabolites, lipids, and proteins/peptides are widely available in the complex extracellular fluid of tears secreted from lacrimal glands, ocular surface epithelial cells, goblet cells, and blood [95]. Through electrochemical biosensor applications, these complex extracellular fluids can be measured for desirable health monitoring analyses [59,82,95,96]. As a natural choice, a contact lens can integrate an amperometric glucose sensor to analyze the tear. Firstly, the sol-gel titania film is applied to immobilize GOx. Next, Nafion is used to decrease interference from the other analytes in the tear to result in a glucose sensor with a fast response of 20 s and high sensitivity of 240 μA/(cm^2^·mM). Integrating the sensor with power supply and wireless signal transduction to a remote electronic device further provides a wireless sensor. The contact lens biosensor is used to wirelessly monitor tear glucose in a rabbit ranging from 0.03 to 5.0 mM [97]. The estimated basal tear glucose of 0.11 mM is shown to have a delay of 10 min from the blood sugar level. Without using enzymes, modifying the electrodes with CuO microparticles from inkjet printing leads to an enzyme-free glucose tear sensor with a high sensitivity of 850 μA·mM^−1^·cm^−2^ and a low limit of detection of 2.99 μM [84]. Besides, ocular contact lenses should not obstruct the field of vision. Thus a highly transparent, multifunctional glucose sensor utilizing graphene and its hybrid with metal nanowires on an actual ocular contact lens is developed [98,99]. With a stretchability of 25% and high transparency of >91%, the sensor has demonstrated its reliable operation through both in vitro and in vivo tests by using a bovine eyeball and living rabbit, respectively. Without the need for direct eye contact, integrating wearable sol-gel tear biosensor on an eyeglasses nose-bridge pad connected to eyeglasses to collect and analyze tear can enable non-invasive monitoring (Figure 3b) [93].

### 3.3. Sweat Analysis

Compared to saliva and tear, sweat that contains abundant biochemical compounds can be monitored from a wider range of locations on the human body. With a temperature sensor for internal calibration, analysis of sweat with a constant flow yields simultaneous and selective measurements of metabolite (e.g., lactate) and electrolytes (e.g., pH, Na^+^) [100]. The trace metal in sweat can also be detected by a wearable amperometric biosensor. This biosensor consists of an Ag/AgCl pseudo-reference, counter, and carbon working electrode modified by Nafion and bismuth for Zn detection. It owns a sensitivity of 23.8 μA∙mL/μg and a limit of detection of 0.05 μg/mL [60]. After preparing patterned Au nanosheets (AuNS) on a stretchable silicon substrate by filtration, deposition of carbon nanotubes (CNTs) is followed by coating of CoWO_4_/CNT (of polyaniline/CNT) nanocomposites on the electrode to result in a skin-attachable electrochemical biosensor for detecting glucose (or pH) in sweat (Figure 3c) [85]. Besides a sensitivity of 10.89 μA/(mM·cm^2^) (or 71.44 mV/pH) for glucose (or pH), the sensors are also stable in air for 10 days and against mechanical deformation with a tensile strain up to 30%. As sweat rates could vary with body movements (e.g., running vs. sitting), it is highly desirable for sweat sensors to deconvolute multiple components in the complex mixture of sweat at different rates. 

### 3.4. Interstitial Fluid (ISF) Analysis

Interstitial fluid (ISF) has a similar composition to that of blood. Each contains essential small molecules (e.g., salts, proteins, glucose, and ethanol). Additionally, it can allow for minimally invasive monitoring without the need for blood sampling [101]. By applying a potential difference between two electrodes on the skin surface, reverse iontophoresis extracts ions such as Na^+^ in the ISF to the skin surface [102,103], which has also been used by the GlucoWatch [104]. Combining reverse iontophoresis with an enzyme-based amperometric biosensor results in a flexible tattoo-based epidermal diagnostic device (Figure 3d) [92]. Compared to GlucoWatch, a GOx-modified Prussian Blue transducer at a low applied potential analyzes the ISF glucose extracted by reverse iontophoresis at a low current density. Non-invasive extraction of the ISF from the subcutaneous tissue can also be enabled by a PDMS-based microfluidic system. This system consists of a micro vacuum generator for transdermal ISF extraction, microchambers for ISF collection, micro-pneumatic valves for fluid management, and a microflow sensor for ISF volume measurement with an error <0.05 μL [105]. Combining the microfluidic chip for ISF collection with a three-electrode electrochemical glucose sensor further leads to a continuous glucose monitoring microsystem [106]. The resolution of glucose measurements is improved by decorating graphene and Au nanoparticles on the working electrode. By providing a composite nanostructured surface, the sensor with the decoration can capture glucose ranging from 0 to 162 mg/dL with a limit of detection of 1.44 mg/dL. 

## 4. Wearable Electrochemical Biosensors Based on Flexible and Stretchable Materials and Structures

### 4.1. Temple and Non-Template Fabrication Methods

**Figure 4:**
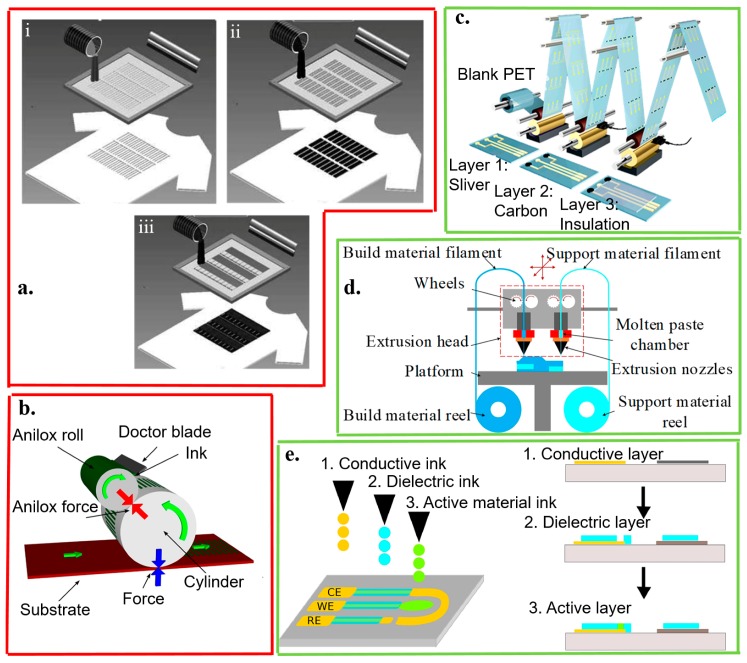
Template- (red boxes) and non-template (green boxes) fabrication approaches. The template fabrication approaches include (**a**) screen printing, (**b**) flexographic process, and (**c**) gravure printing. The screen printing explores (i) laser-cut stainless steel or chemically-etched polymeric mesh-screen stencils for patterning (ii) the Ag/AgCl reference and (iii) working/counter electrodes with carbon- or metal-based ink containing recognition elements overlaid on the Ag/AgCl conductor; reproduced with permission from [50], Copyright 2013, Wiley-VCH. In the flexographic process, the surface of the anilox roller consists of engraved cells. The doctor blade helps to remove excess ink from the anilox; reproduced with permission from [112], Copyright 2017, Elsevier. By using a rotary printing press in the gravure printing, the image is engraved onto a cylinder; reproduced with permission from [113], Copyright 2018, American Chemical Society. The non-template fabrication approaches include (**d**) 3D printing and (**e**) inkjet printing. In extrusion-based 3D printing, the build material filament is heated, melted, and extruded in the nozzle. Each layer is deposited on the previous layer to form the designed 3D structure; reproduced with permission from [114], Copyright 2016, Wiley-VCH. In inkjet printing, layers of conducting and dielectric materials are injected, patterned, and stacked on a substrate; reproduced with permission from [115], Copyright 2015, Elsevier.

Several novel fabrication methods have been developed for flexible and stretchable electrochemical biosensors [48,107,108]. Lithographic approaches (e.g., thin-film deposition and etching, photolithography, and ion-beam lithography) can be used to reproducibly fabricate high-performance devices (e.g., H_2_O_2_ sensors [108] and RNA sensors [109]). However, their attractive attributes come at a high cost due to the cleanroom setup, multiple equipment acquisitions, complex processes, and the unique materials required [108,109,110]. Recent developments in advanced materials such as new ink formulations help promote the development of various printing technologies (Figure 4) [111,112,113,114,115]. These new developments partially address the challenges in high-cost lithographic approaches that also have compromised performance on rough or textured surfaces. Both the leading template and non-template-based printing technologies for fabricating electrochemical biosensors are summarized and compared (Table 3).

Because the screen printing technique is low cost and easy to scale up for mass production of electrochemical biosensors with the favorable electroanalytical performance [116,117], it results easily in low-cost fabric/textile-based electrochemical biosensors [118]. Firstly, the conductive ink (e.g., Ag/AgCl as a reference electrode) is applied as an underlayer on the textile. Next, carbon or metal-based ink containing the recognition element is overlaid on the underlayer to work as the working electrodes (Figure 4a) [50]. The spatial resolution and electrical performance of printed electrodes hinge on ink formulation. For instance, the ink with a nearly defect-free graphene oxide derivative can be printed to result in high-resolution lines with a width below 100 μm, a thickness of 3 μm, and a sheet-resistance below 1 Ω/sq [119]. 

The other template-based printing methods also include flexography and gravure printing, where the ink is transferred to the substrate from a raised (flexography) or engraved (gravure) pattern on a roll. In flexography printing (Figure 4b) [112], the ink is first transferred from a bath to an anilox roll. The anilox roll contains millions of tiny divots to take up the ink, which can bring the anilox roll in contact with the printing cylinder. The ink is then transferred to the surface of the target substrates. While the ink is on the ridges of the pattern on the printing cylinder in flexography [87,94,95], gravure printing relies on impressing the film into the cavities of the roll where the ink resides (Figure 4c) [113]. Both these two printing methods are intrinsically robust and can enable large-area manufacturing [113,119,120,121,122,123,124].

In addition to the template-based printing methods, non-template-based printing technologies have been developed because of their higher customization design and lower price for small-scale manufacturing. Non-template-based printing methods rely on dispensing the given technology. These technologies include the use of gas or pressurized air (pneumatic), the use of piezoelectric material in the setup (piezoelectric), the use of aerodynamic focusing (aerosol jet), the driving of ink by an electric field (electrohydrodynamic), and the heating the material (thermal). [125,126]. As a representative non-template-based printing method, extrusion-based 3D printing applies the ink filament through a heated nozzle onto the substrate via a computer-controlled motion stage (e.g., three-axis) to manufacture a fully 3D-printed electrode (Figure 4d) [114]. By contrast with extrusion-based 3D printing that often requires high viscosity (>300 k cP) in the inks, inkjet printing explores the low viscosity (10–20 cP) inks to help ink transfer (Figure 4e). However, low-viscosity inks suffer from small filler loading [115,125]. 

While the electrochemical performance of biosensors hinges on the specific materials of the electrodes in the sensor, the substrate material affects their mechanical properties, which is related to the level of comfort and may also result in a change in their electrochemical performance. The commonly used substrate materials include the textile, flexible thin films (e.g., paper, plastic, and tattoo-like thin films), and stretchable thin films. Table 4 summarizes the representative electrochemical biosensors based on different substrate materials.

### 4.2. Textile-Based Biosensors

Being flexible and widely used in our daily life, textiles such as wool, cotton, and nylon have been extensively exploited as the substrate for integrating various electrochemical biosensors [150]. Early investigations of textile-based chemical biosensors rely on optical systems for bio-sensing. This is through the utilization of a light source and detector [35,36,37]. However, the required optical sensors are sophisticated and high in cost. By leveraging the recently developed fabrication techniques, simple textiles-based electrochemical biosensors have been obtained to withstand repeated bending cycles. The influence of textile substrates (e.g., cotton, polyester, and widely used breathable and water-proof GORE-TEX^®^ fabric) on the sensing performance of nitroaromatic explosives has been investigated when integrated screen-printed electrodes on different textile substrates [118]. The adhesion at the electrode/textile interface is demonstrated to be robust against cycles of laundry washing and mechanical deformations. 

**Figure 5:**
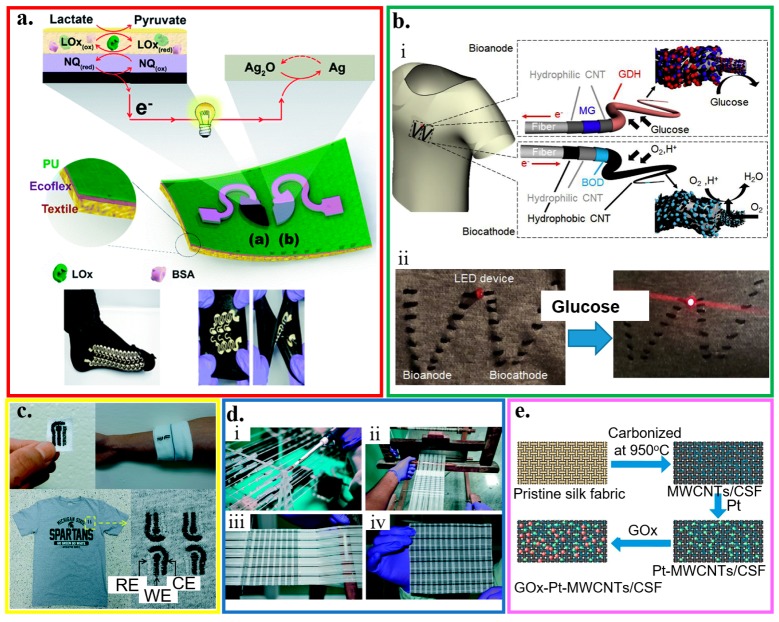
Fabric-based electrochemical sensors. (**a**) Optical images of the designed stencil and its use for printed stretchable devices through a screen printing process; reproduced with permission from [54], Copyright 2016, The Royal Society of Chemistry. (**b**) (i) Schematic of enzyme/carbon nanotube (CNT) composite fibers woven on a textile cloth. The anode and cathode fibers were prepared by modifying multi-walled CNT-decorated carbon fibers with glucose dehydrogenase and bilirubin oxidase, respectively. (ii) Illumination of a light-emitting diode (LED) device consisting of a charge pump IC, capacitor, and red LED connected to enzymatic power fibers upon dropping a glucose solution on a cloth; reproduced with permission from [151], Copyright 2019, Elsevier. (**c**) Embroidered electrochemical sensors fabricated on textile, cotton gauze, and cotton t-shirt; reproduced with permission from [133], Copyright 2016, The Royal Society of Chemistry. (**d**) Manufacturing of fabric-based electrochemical sensors: (i) custom-made yarn coating instrument, (ii) handloom used to weave the sensors, (iii) woven patches on the loom, and (iv) a woven array of 90 (15 × 6) sensors; reproduced with permission from [152], Copyright 2015, The Royal Society of Chemistry. (**e**) Schematic of processes to prepare the glucose sensor based on the carbonization of textiles; reproduced with permission from [153], Copyright 2018, Elsevier.

Applying the technique of screen printing yields a highly stretchable textile-based biofuel cell to analyze sweat metabolites (Figure 5a) [54]. The glucose (or lactate) biofuel cell with single-enzyme and membrane-free configurations could generate a maximum power density of 160 (or 250) μW cm^−2^ with an open-circuit voltage of 0.44 (or 0.46) V. Enzymatically oxidized on the anode, the biofuel (e.g., glucose) releases electrons that are accepted by the cathode. The generated power can operate on human-body sweat to provide a self-powered response. Intrinsically stretchable inks (i.e., CNTs dispersed in Ecoflex) and stretchable structure of the serpentine electrode are employed in the device. The resulting self-powered devices can exhibit a high stretchability (a tensile strain of 100%) and endure a stable performance upon repeated (>100 times) strains. An alternative wearable, high-power biofuel cell explores a glucose-oxidizing glucose dehydrogenase as anode and an O_2_-diffusion bilirubin oxidase as a cathode on a textile cloth (Figure 5b) [151]. Two types of CNT layers are used to improve the performance of the anode and cathode: an acid-treated hydrophilic CNT layer for coating of the mediator and enzyme, and polytetrafluoroethylene (PTFE)-based hydrophobic CNT layer for adequate oxygen diffusion by forming a microporous layer. Owning to the stretchable material and structure, the maximum power density can still maintain 216 μW/cm^2^ at an output voltage of 0.36 V for glucose of 200 mM even upon deformation (e.g., S-shape). By using a series connection of four biofuel cell, power can be generated with an open-circuit voltage of 1.9 V to illuminate a light-emitting diode (LED) on the cloth.

Compared to the integration of electrodes on the textile with a conventional screen-printing method that results in ink waste, embroidery and yarn coating use only as much reagent and ink as required. Conductive threads with the immobilized enzyme can be embroidered into textiles to serve as the working, counter, and reference electrodes in an electrochemical biosensor with three-electrode configuration to quantitatively analyze biofluid samples (Figure 5c) [133]. The electrodes with customized geometries at specific locations on a garment can be quickly fabricated by using a computerized embroidery machine. The hydrophilic nature of most threads in embroidered sensors can help quickly absorb liquids to facilitate sample loading for improved automation. The embroidered electrochemical biosensor exhibits a stable performance with a marginal decrease of 9% in the signal after 100 bending cycles. Multiplexed measurements of different targets (e.g., glucose and lactate) can also be achieved by using selective assays to each target (e.g., a glucose assay and a lactate assay). With negligible signals from the non-specific analytes, the glucose (or lactate) assay only selectively responds to the glucose (or lactate) with a significant response and a high signal-to-noise ratio of 3.2 (or 4.1) at a concentration of 5 mM (or 12.5 mM). Similar to embroidery, yarn coating allows the use of textile weaving with a wide variety of yarn materials, weaving styles, and looms to create electrochemical biosensors with various properties. For instance, silk yarns coated with conducting inks can be handloom-woven as electrodes into patches of fabric to create arrays of sensors, which are then laminated, cut, and packaged into individual sensors. By using the sensor consisting of four electrodes with one working electrode for hemoglobin and one working electrode for glucose, a multiplexed array can simultaneously detect glucose and hemoglobin from the blood samples (Figure 5d) [152]. While the use of an analyte-specific enzyme (i.e., glucose oxidase) on the working electrode provides a highly selective glucose detection, the carbon electrode with differential pulse voltammetry (DPV) detects hemoglobin with no significant interference from glucose. The shared counter and reference electrodes in the multiplexed sensor also help reduce the cost. 

As an alternative to the weaving/embroidery of conducting fabric or integration of other conductive materials, the carbonization of textiles coated with nanomaterials presents another route to the creation of the electrochemical biosensors. In a representative example, silk fabrics coated with multi-walled carbon nanotubes to fully use the space and strengthen the interconnection are first carbonized from hydrophilic to relatively hydrophobic. Decorating the resulting structure with Pt microspheres (or glucose oxidase, GOx) enables the detection of H_2_O_2_ (or glucose) (Figure 5e) [153]. The glucose sensor obtained has a sensitivity of 288.86 μA/(mM cm^2^) in a relatively good linear range from 0 to 5 mM. As the intimate contact between the sensor and skin is highly desirable to allow for the precise measurement of the target analyte, tight-fit clothing has been explored. As a representative example, textile-based amperometric biosensors are integrated on an elastic waistband of common underwear for direct tight contact [31]. However, the level of comfort is significantly compromised. Additionally, measurements of analyte concentrations are limited to specific locations that have intimate contact between textile-based devices and the skin [38]. Therefore, thin-film sensors based on the other flexible and stretchable substrates have been developed to address some of these concerns.

### 4.3. Flexible Thin-Film Biosensors

#### 4.3.1. Paper-Based Biosensors

**Figure 6:**
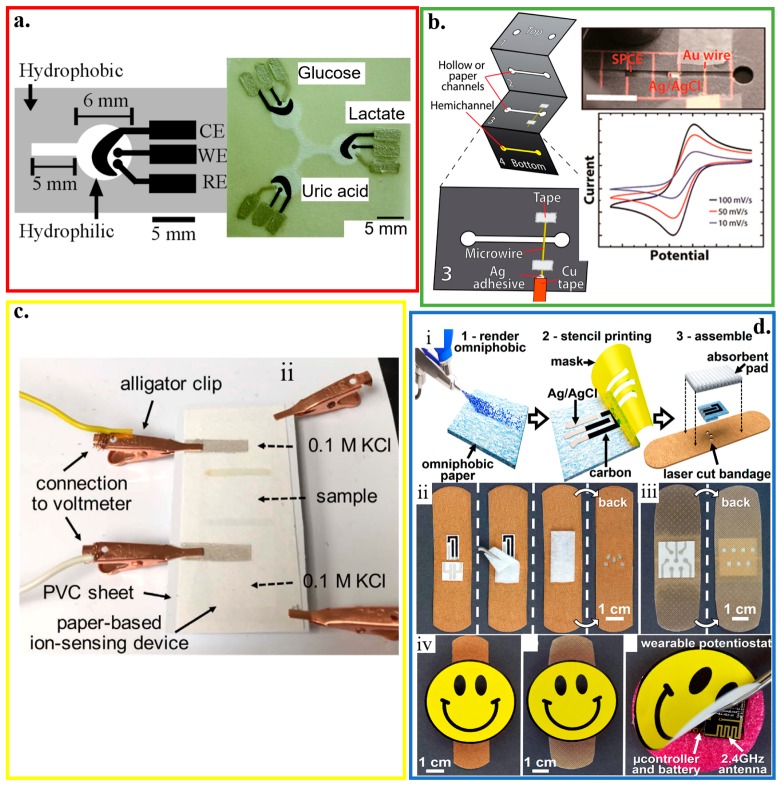
Paper-based electrochemical biosensors. (**a**) Design and optical image of the electrochemical detection cell for paper-based microfluidic devices. WE/CE: working/counter electrode (carbon ink); RE: reference electrode (silver/silver chloride ink); reproduced with permission from [55], Copyright 2009, American Chemical Society. (**b**) Paper-based microelectrochemical devices with electrodes based on conductive wires, inlet/outlet in the 1^st^ layer, and two stacked channels in the other 3 layers; reproduced with permission from [161], Copyright 2014, American Chemical Society. (**c**) Optical image of a paper-based ion-sensing platform, with two alligator clips on the left to measure the electromotive force (EMF) and two clips on the right for balancing; reproduced with permission from [165], Copyright 2016, WILEY-VCH. (**d**) Fabrication and assembly process of omniphobic paper-based smart bandage (OPSB): (i) Schematic of the fabrication of OPSBs: (1) After spraying RFSiCl3 of 2% in IPA to render Whatman #1 paper omniphobic, (2) flexible carbon and Ag/AgCl electrodes are patterned through stencil printing, followed by (3) laser-cutting the adhesive layer of the bandage for creating openings to interface with the wearable potentiostat. Placing the paper-based sensors between the adhesive layer and the absorbent pad of the commercial bandages assembles the OPSBs, which can monitor (ii) uric acid and pH levels in open wounds, as well as (iii) the early detection of pressure ulcers. (iv) shows the packaging of the electronics in the rechargeable, wearable potentiostat; reproduced with permission from [166], Copyright 2018, Elsevier.

Being flexible, foldable and rollable, widely available, inexpensive, lightweight, and hydrophilic, paper can be readily and rapidly modified with biomolecules and nanomaterials for electrochemical-sensing applications. The high porosity of cellulose in the paper also allows for solution transport through capillary forces. It can serve as an autonomous microfluidic pumping system without a need for external pumps [154]. The paper-based bioanalytical devices use capillary forces to drive the lateral flow of a liquid sample (i.e., lateral flow immunochromatographic assays or lateral flow tests) [155,156]. Based on the generated color, lateral flow tests can provide qualitative or semi-quantitative information [157]. Quantitative measurements can also be obtained with electrochemical paper-based analytical devices (ePADs) that use the photolithography to create microfluidic channels on the filter paper (Figure 6a) [55]. The Ag/AgCl ink is first applied as the reference electrode and conductive pads. Next, screen-printing carbon ink that contains Prussian Blue results in the working and counter electrodes. Spotting the analyte-specific enzymes (e.g., GOx, lactate oxidase or LOx, and uricase) helps determine the concentration of glucose, lactate, and uric acid, with a limit of detection of 0.21 ± 0.02 mM, 0.36 ± 0.03 mM, and 1.38 ± 0.13 mM, respectively. As a close distance between the working and reference electrodes minimizes the effect from uncompensated resistance between the two electrodes, screen printing that requires a stencil to pattern the electrodes limits the achievable maximum resolution [157,158,159,160]. 

Although the shape of electrodes is well-defined with a patterned screen or stencil, they often suffer from poor electrical properties and irreproducible surface chemical properties. As an alternative, prefabricated Au microwires and carbon fibers, along with their meshes, can be exploited as electrodes in a paper-based device with a multilayered structure created by the folding principles of origami (Figure 6b) [161]. When a large surface area is desirable, mesh electrodes are preferred over their wire counterparts to provide a larger surface area for the immobilization of bioprobes. Without using a patterned screen or stencil, portable writing tools such as lead pencils can directly draw electrodes with the desired geometry on paper to create electrochemical devices [162]. After pressurization, the mixture consisting of carbon powder as a conductive material, sodium bentonite as a binding agent, and sodium silicate as a hardening agent in thin rods can be inserted in commercial lead holders to facilitate drawing on paper. 

With precise control of ink droplet volume, inkjet printing has also been explored to fabricate paper-based electrochemical biosensors [163]. In the inkjet printing paper-based electrochemical biosensor with a three-electrode configuration [164], the electrochemical deposition of Ag/AgCl on an inkjet-printed Ag nanoparticle pattern serves as the reference electrode. After inkjet-printing of nanoparticle-based gold working and counter electrodes, electropolymerized polyaniline (or GOx entrapped poly-3,4-ethylenedioxythiophene) films on the surface of the working electrode enable selective sensing of pH (or glucose), which has comparable performance with their commercial counterparts. Applying inkjet printing can also integrate a potentiometric cell into a piece of filter paper to form a paper-based ion-selective platform (Figure 6c) [165]. This device uses a hydrophilic high-capacity ion-exchange membrane and a valinomycin-doped ion-selective electrode (ISE) membrane embedded into the paper. It achieves highly selective sensing of Cl^−^ and K^+^ with a sensitivity of 57.4 ± 0.5 mV/decade and 53.3 ± 0.7 mV/decade, respectively.

Integrating paper-based biosensors with other platforms such as a commercial bandage may open additional opportunities, such as a smart bandage. The screen-printed conductive inks are embedded into commercial bandages. The developed omniphobic paper-based smart bandage (OPSB) with a lightweight (~8 g) sensor is capable of measuring pH and uric acid in open wounds and pressure ulcers for chronic wound monitoring (Figure 6d) [166]. Taken together with a wireless communication module, the wearable OPSB can simultaneously quantify pH and uric acid levels at the wound site to wirelessly inform the user of wound status [167] at low-cost (~$18).

#### 4.3.2. Plastic-Based Biosensors

**Figure 7:**
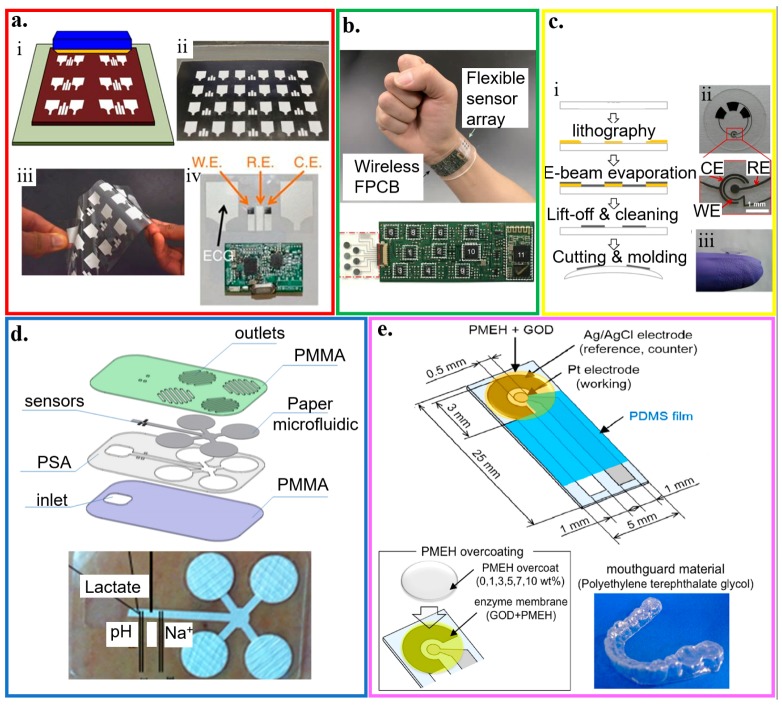
Plastic-based wearable electrochemical biosensors. (**a**) (i) Schematic and (ii) optical image of the fabrication of the electrochemical sensor through screen printing, as well as (iii) its flexibility demonstration and (iv) integration with the wireless electronics; reproduced with permission from [56], Copyright 2016, Macmillan Publishers Limited. (**b**) Optical image of a wearable flexible integrated sensing array (FISA) on the wrist of a subject; reproduced with permission from [22], Copyright 2016, Macmillan Publishers Limited. (**c**) (i) Fabrication process and (ii,iii) optical images of a lactate sensor on the contact lens with sensing structure on (ii) a flat substrate and (iii) a completed contact lens held on a finger; reproduced with permission from [82], Copyright 2012, Elsevier. (**d**) Exploded view and optical image of the microfluidic chip with microneedles for sweat collection and analysis. The sensors were placed inside the microfluidic channel that could draw a continuous flow of sweat; reproduced with permission from [171], Copyright 2017, Elsevier. (**e**) Schematic and optical image of the glucose biosensor on the polyethylene terephthalate glycol (PETG) mouthguard, with Pt and Ag electrodes formed by a sputtering process; reproduced with permission from [172], Copyright 2016, Elsevier.

Compared to paper, plastic substrates (e.g., polyester family, polyethylene naphthalene, polytetrafluoroethylene, and many others) have sufficient thermal stability, low coefficient of thermal expansion, and structural resiliency against deformation [125]. By using the widely reported flexible polyester with a thickness of 50 μm as a representative example [82,168,169,170], a three-electrode amperometric lactate biosensor is fabricated along with a bipolar electrocardiogram sensor in a wearable hybrid sensing system (Figure 7a) [56]. The three amperometric electrodes are also separated from the Ag/AgCl electrocardiogram electrodes via a printed hydrophobic layer to increase sensor stability and signal-to-noise ratio. The working electrode of the lactate biosensor is coated with LOx-modified Prussian Blue as a biocatalytic layer for selective detection of lactate, which has a sensitivity of 96 nA/mM in a linear range for the lactate concentration from 0 to 28 mM. As a representative example in the polyester family, the polyethylene terephthalate (PET) substrate has also been widely explored for the thin-film sensors. For instance, a mechanically flexible and fully integrated sensor array can also be embodied on the PET substrate for multiplexed in situ perspiration analysis (Figure 7b) [22]. The integrated sensor array can simultaneously and selectively measure sweat metabolites (glucose and lactate) and electrolytes (sodium and potassium ions), along with the skin temperature to calibrate the response of the other sensors. Molding a 100 μm-thick PET into a contact lens shape allows for the integration of sensors to detect lactate with an average sensitivity of ∼53 μA/(mM cm^2^) within the linear range from 0 and 1 mM and a relatively fast response time of 35 s (Figure 7c) [82].

A flexible hybrid poly(methyl methacrylate) (PMMA)/paper microfluidic platform with fully integrated sensing can simultaneously monitor lactate, Na^+^, and pH for on-body testing of human sweat (Figure 7d) [171]. A continuous flow of sweat is collected by microneedles with an array of Pt and Ag wires (50 μm diameter) and transported in a paper microfluidic channel. The Pt and Ag wire microneedles also serve as the working and reference electrodes for the lactate/Na^+^/pH sensors. Before drop-casting LOx on the working electrode in the amperometric-based lactate sensor, a semipermeable copolymer membrane (sulfonated polyether ether sulphone-polyether sulphone, SPEES/PES) is applied to achieve high selectivity, following by a coating of an outer polyurethane layer. The pH sensor relies on a pH-sensitive iridium oxide (IrOx) membrane to yield a sensitivity of 71.90 ± 0.8 mV/unit. And the potentiometric Na^+^ sensor exploits a bilayered structure with polyvinyl chloride (PVC) membrane on a poly(3,4-ethylenedioxythiophene) (PEDOT) polymer to result in a sensitivity of 56 ± 1 mV/unit.

Because of its strong adhesion to Pt and Ag, PET glycol (PETG) is used as the platform (e.g., PETG mouthguard) to integrate Ag/AgCl reference electrode and Pt working electrode with GOx immobilized by poly (MPC-co-EHMA) (PMEH) for monitoring saliva glucose (Figure 7e) [172]. By using a 1.0 wt% PMEH overcoat and an electrode surface area of 16.8 mm^2^, optimized glucose measurement in artificial saliva with a phantom jaw is achieved with a stable response within ~60 s and good sensitivity for the glucose concentration from 0.05 to 1.0 mM.

#### 4.3.3. Temporary Tattoo-Based Biosensors

**Figure 8:**
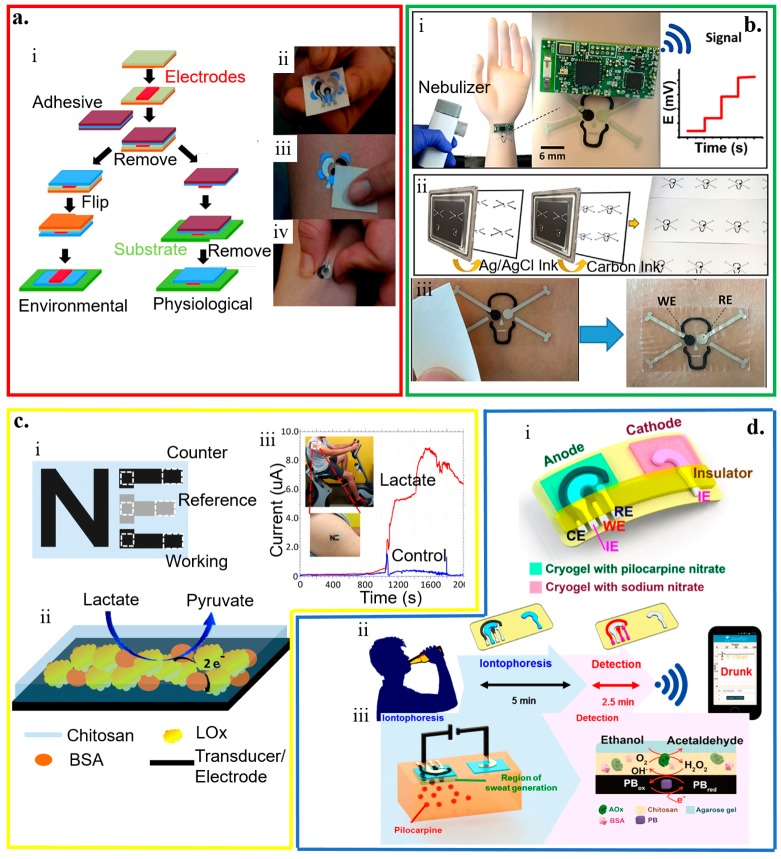
Temporary tattoo-based wearable electrochemical biosensors. (**a**) (i) Schematic and (ii–iv) optical images of the processes to prepare temporary transfer on-skin tattoo-like electrochemical sensor; reproduced with permission from [57], Copyright 2012, The Royal Society of Chemistry. (**b**) Tattoo-like biosensor for detecting nerve agents: (i) Image of the integrated potentiometric biosensor system placed on the mannequin for wireless signal transmission with schematics to show (ii) sensors printed on the tattoo paper and optical images to show (iii) sensors transferred to the skin after removal of the protective layer; reproduced with permission from [175], Copyright 2018, Elsevier. (**c**) (i) Schematic illustration of a three-electrode “NE” tattoo-like electrochemical sensor to detect epidermal lactate, with its (ii) working principle and (iii) demonstration of sweat lactate monitoring during cycling exercise; reproduced with permission from [60], Copyright 2013, American Chemical Society. (**d**) Tattoo-based transdermal alcohol sensor. (i) Schematic diagram of an iontophoretic-sensing tattoo-like device for transdermal alcohol sensing, as well as schematic diagrams to show (ii) its wireless operation and (iii) constituents in the iontophoretic system; reproduced with permission from [46], Copyright 2016, American Chemical Society.

Tattoo-like electrochemical biosensors are attractive because of their intimate contact with the human skin without causing much discomfort on the body [2,173]. In fabricating temporary transfer tattoo-based electrochemical biosensors (Figure 8a) [57], the electrode designed in red with active ink materials (e.g., carbon and Ag/AgCl reinforced with carbon fibers) is first patterned by screen printing on paper (orange) coated with the release agent (olive). After applying the adhesive sheet (blue) with a protective coating (maroon) on the printed sensor, removing the protective sheet and flipping the layers can apply it onto the skin (green). Removing the release agent-coated paper then exposes the sensor. Before removing the protective sheet, the release agent-coated paper can be removed to allow direct contact of the electrode to the skin. In addition to favorable electrochemical properties as opposed to the electrodes from the conventional screen printing, the resulting sensor also exhibits robust performance against various deformation modes (e.g., pinching, bending, and twisting), with promising applications as potentiometric and amperometric sensors [60,147,148,173,174,175,176,177].

The accuracy of conventional potentiometric biosensors hinges on a stable and reproducible potential of the liquid junction at both reference and working electrodes. However, the leakage of the solution becomes a concern [38]. By exploring the concept of all-solid-state electrodes [147,148], a wearable potentiometric all-solid-state biosensor (without inner liquids) is developed for real-time on-body monitoring of nerve agents simulant diisopropyl fluorophosphate (DFP) (Figure 8b) [175]. The enzymatic hydrolysis of DFP by the enzyme of organophosphate hydrolase (OPH) results in proton release. The resulting pH change captured by the skin-worn potentiometric pH-sensing transducer directly correlates to the DFP in the liquid and gas phases. The device in the design of a “skull face’’ layout consists of one ‘eye’ from an Ag/AgCl reference electrode and the other ‘eye’ from a printed carbon working electrode coated with polyaniline (PANI). The sensor can detect the DFP in the liquid phase with a limit of detection of 10 mM and a stable response in less than 20 s for the concentration from 10 to 120 mM. The detection of the DFP in the vapor phase is slightly longer but still within 30 s. With the same limit of detection of ~10 mM, the sensor response increases linearly as the vapor concentration increases from 20 to 120 mM.

By using a mediated LOx working electrode in an amperometric biosensor, the printed temporary-transfer tattoo electrochemical biosensor enables real-time lactate sensing (Figure 8c) [60]. The LOx working electrode is prepared by first tethering the LOx enzyme on the surface of the printed tattoo electrode functionalized with tetrathiafulvalene and multiwalled carbon nanotubes. Next, a biocompatible chitosan overlayer is coated. The resulting sensor exhibits a high sensitivity of 10.31 μA/(mM cm^2^), a very good specificity with negligible responses from interfering agents (e.g., ascorbic acid, uric acid, glucose, and creatinine) of less than 5%, and a highly linear response for the lactate concentration ranging from 1 to 20 mM.

Combining reverse iontophoresis (RI) to extract interstitial fluid (ISF) glucose to the skin surface results in a tattoo-based noninvasive glucose monitoring system [61]. This enzymatic amperometric biosensor has a similar principle of the GlucoWatch glucose sensor. The device system has one pair of the anodic and cathodic contingents, with each consisting of a group of working, counter, and reference electrodes encompassed by an additional Ag/AgCl RI electrode for efficient extraction of ISF. The glucose tattoo sensor exhibits a sensitivity of 23 nA/μM and a limit of detection of 3 μM, and a linear response range from 0 to 100 μM. Exploiting two iontophoretic electrodes (anode and cathode) with three amperometric sensing electrodes (working, reference, and counter electrodes) in the anode compartment can also yield a wearable alcohol sensor system (Figure 8d) [46]. By delivering the pilocarpine drug from the anode compartment, iontophoretic electrodes induce the sweat in the anode region for the alcohol analysis with a high sensitivity to detect ethanol (0.362 ± 0.009 μA/mM). Because of the use of alcohol oxidase, the alcohol sensor demonstrates negligible interferences from glucose, uric acid, lactate, ascorbic acid, and creatine.

### 4.4. Stretchable Thin-Film Biosensors

Flexible thin-film biosensors can withstand a mechanical strain before fracture (e.g., <1% for paper and <5% for plastic substrates). However, opportunities still exist for electrochemical biosensors when the applied strain from various loading conditions exceeds the fracture limit [177,178,179]. Additionally, the flexible thin-film biosensors cannot conform to the textured surface of the skin at various locations on the human body. Imparting stretchable characteristics in the devices represents a simple yet effective strategy to integrate them on the non-developable surfaces (i.e., non-zero Gaussian curvature) of the skin [15,180,181,182,183]. The stretchable devices can be realized by exploiting either intrinsically stretchable materials or stretchable structures. The stretchable structures [184,185] applied for electrochemical biosensors include wavy thin-film structures [186,187], serpentine structures [57,129,188,189], mesh structures [190,191], island-bridge structures [192,193], among others.

**Figure 9:**
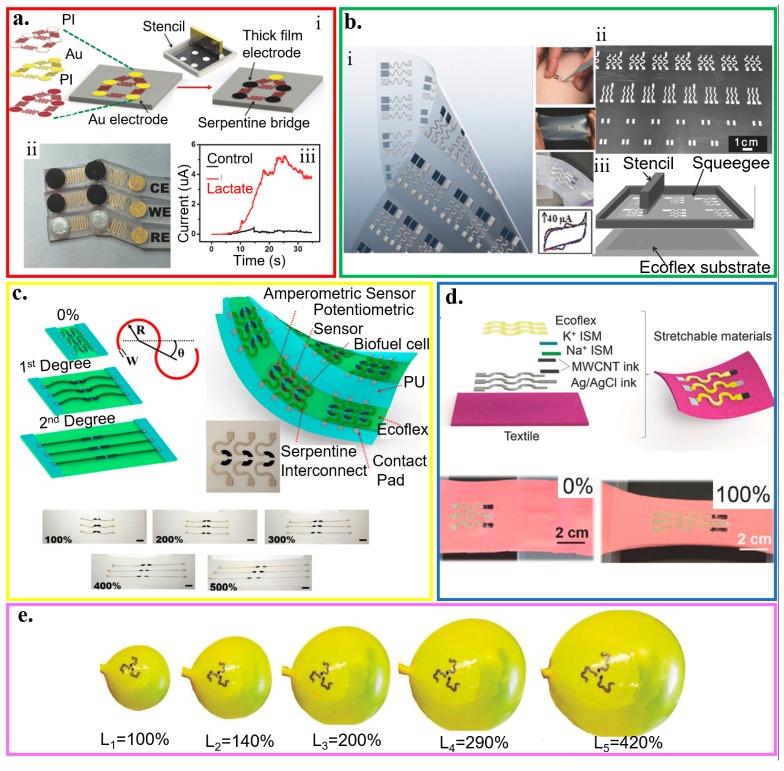
Wearable electrochemical biosensors based on stretchable structures. (**a**) (i) Schematic of the fabrication processes that merge lithographically fabricated thin- and printed thick-film for a hybrid, stretchable electrochemical sensor, along with (ii) an image of the stretchable lactate sensor (working, reference, and counter electrodes) and (iii) its demonstration for real-time on-body amperometric evaluation of lactate levels from a subject with (red) and without (black) enzyme modification; reproduced with permission from [192], Copyright 2017, Wiley-VCH. (**b**) (i) Optical image and (ii) fabrication process of different patterns of interconnects that connect electrodes and contact pads by printing inks through a stencil with (iii) a schematic of the screen printing; reproduced with permission from [188], Copyright 2015, Wiley-VCH. (**c**) Combining stretchable serpentine structures with intrinsically stretchable nanomaterial-based inks results in highly stretchable electrochemical sensors; reproduced with permission from [58], Copyright 2015, American Chemical Society. (**d**) Schematic representation and optical images of the electrochemical sensors with stretchable structures applied on stretchable textile substrates upon a tensile strain of 100%. The sensor was fabricated by screen printing of stretchable Ag/AgCl ink, stretchable CNT ink, and Ecoflex layer, followed by surface modification of ion-selective membrane at specific locations; reproduced with permission from [128], Copyright 2016, Wiley-VCH. (**e**) Series of optical images to show different inflation levels of the expandable electrochemical device; reproduced with permission from [189], Copyright 2016, Wiley-VCH.

With the “island–bridge” design, the rigid electrodes (i.e., islands) from screen printing are connected by serpentine interconnects (i.e., bridges) from lithography on an elastomeric substrate (Figure 9a) [192]. Without a specific requirement for the material in the rigid islands, the electrodes can then be prepared with a wide range of functional materials such as printable inks (e.g., Ag/AgCl, enzyme-loaded Prussian blue, and carbon inks). The bridges can also be replaced by other stretchable structures to provide a variety of different stretchable layouts. By selecting ferricyanide and dopamine as target analytes, the electrochemical biosensor exhibits a negligible response change even for a repeated biaxial strain of 75%. Applying the structure in a lactate biosensor measures the real-time epidermal sweat lactate from the linear current response to the lactate level (Figure 9a(ii,iii)). After the perspiration of the subject at ~900 s, increasing the cycling intensity results in an increase in the sweat lactate level, as evidenced by the rising current. 

Without using the lithographic approach for the bridges, the serpentine structures (arc angle of 180°) that connect electrodes and contact pads can also be created by screen printing of conducting inks with tailored elastomer and surfactant through a custom-designed stencil (Figure 9b) [188]. The detection of ferricyanide with cyclic voltammetry shows a minimal change in the peak current before and after ten fatigue cycles (stretching to 100% and then back to 0) for a total of 50 repetitions. Free-standing serpentine structures with an optimized arc angle can also be combined with intrinsically stretchable nanomaterial-based inks to result in a highly stretchable CNT-based electrochemical biosensor (tensile strain up to 500%) (Figure 9c) [58]. After the free-standing serpentine interconnects fully unwind upon stretching, further applied tensile strain leads to an increase in the resistance of the intrinsically stretchable CNT inks. The electrochemical biosensors with stretchable structures can also be applied to stretchable textile substrates. Combining polyurethane (PU)-based ion-selective membranes with CNT binder inks along with Ecoflex-containing Ag/AgCl inks printed in serpentine pattern results in a highly stretchable textile-based potentiometric biosensor that can withstand a tensile strain of 100% (Figure 9d) [128]. The PU matrix provides the biocompatibility and resistance against mechanical stress/strain that is lacking in ion-selective membranes based on PVC matrices. By exploiting adsorptive stripping voltammetry (AdSV) to assay trace amounts of species with interfacial adsorption on the working electrode, similar stretchable electrochemical biosensors can also detect explosive compounds. This sensor consists of the Ag/AgCl–Ecoflex reference electrode and the CNTs-PU working/counter electrodes. The sensor can detect 2,4,6-trinitrotoluene, 2,4-dinitrotoluene, and hydrogen peroxide with negligible changes in response to extreme multiaxial and bending deformations from the inflation and deflation of a balloon (>400% increase in the balloon area) (Figure 9e) [189].

## 5. Conclusions and Future Perspectives

In this mini-review, we have briefly summarized the recent development of flexible and stretchable electrochemical biosensors for personal healthcare, which has experienced remarkable growth over the past few decades. Integrating these sensors with affordable and advanced wireless modules [194,195,196] results in functional devices that can continuously detect and analyze biofluids such as saliva, tear, sweat, and interstitial fluid. Despite the significant strides achieved in the field of electrochemical biosensors, several challenges still exist before their wide adoption in practical and daily applications. First of all, effective sampling of biofluids from the body is crucial to ensure accurate sensing results, necessitating the need for a biofluid sampling and collection module in the system [197,198,199]. It is also highly desirable to improve the sensing performance of wearable electrochemical biosensors. While significant efforts have been devoted to the development of highly sensitive sensors, their response to various interfering factors in the complex biofluids cannot be ignored, especially when a trace amount of target analyte is present. The deconvolution of multiple components from a mixture by a high-density array represents a promising approach to address such a challenge [11]. 

The wearable electrochemical biosensors should also maintain stable performance with minimal interfacial adhesion issues against washing or relatively high temperature. A possible concern from the relatively high temperature is the damage of the sensor in a hot shower [189]. While there are plenty of strategies to achieve a high dry adhesion, robust wet adhesion is of more relevance to the application of wearable electrochemical biosensors. The bioinspired materials (e.g., gelatin-, collagen-, or chitosan-based materials) [200,201,202] have been studied and developed to provide an improved wet adhesion [203]. Exploring these materials can achieve stable binding between functional layers (e.g., electrode and substrate) in the sensors and at the sensor/skin interface, despite the drastic differences in their physical and chemical properties. However, attention still needs to be paid to biocompatibility, tunable adhesion strength, reusability, and compliance [204]. In addition to the bio-integrated wearable devices to sample the biofluids from the skin surface, exploiting the recently developed biodegradable electronics [205,206,207,208,209,210,211,212,213] could open up new opportunities for transient electrochemical biosensors to access biofluids from inside the body. Additionally, real-time monitoring of various biofluid contents from different populations presents an excellent opportunity for big data analytics, which can help accurately inform the health condition and provide in-time treatment [103].

## Figures and Tables

**Table 1:** Summary of the working principles of different electrochemical sensors.

Detection Mode	Transducer	Analytes
Amperometric/voltammetric sensors	Carbon, metal, chemically modified electrodes	Alcohols, glucose, phenols, lactate
Potentiometric sensors	Ion-selective, carbon, metal, glass electrodes	K^+^, Cl^-^, Ca^2+^, Na^+^
Field-effect transistors	Ion-sensitive/enzyme field-effect transistor	K^+^, H^+^
Impedimetric sensors	Interdigitated/metal electrodes	K^+^, helicobacter pylori

**Table 2:** Summary of representative electrochemical biosensors with applications of biofluid analysis.

Biofluid	Platform	Recognition Element	Analyte	Technique	Response Time	Linearity Range	LOD	Detection Sensitivity	Ref.
Saliva	PET	Uricase enzyme	Uric acid	Amperometry	NR (real-time)	0–1.0 mM	NR	2.32 μA/mM in artificial saliva 1.08 µA/mM in undiluted human saliva	[76]
	PB	LOx	Lactate	Amperometry	NR (real-time)	0.1–1.0 mM	NR	0.202 µA/mM in undiluted human saliva	[77]
	Foil	Carbon	(carboxymethyl)lysine	Amperometry	NR (real-time)	0.5–10 μg/mL	0.8 μM	NR	[78]
	polyester	GOx	Glucose	Amperometry	NR (real-time)	0–1.0 mM	5 μM	41.7 μA·mM^−1^·cm^−2^	[79]
	PETG	GOx	Glucose	Amperometry	NR (real-time)	0.1–1.0 mM	NR	NR	[80]
Tear	PET	GOx	Glucose	Amperometry	NR	10 μM–100 mM	5 μM	NR	[81]
				Amperometry	35 s	0–100 μM	50 μM	53 μA·mM^−1^·cm^−2^	[82]
				Amperometry	NR (real-time)	0.1–0.6mM	NR	NR	[83]
	Polyethylene	CuO	Glucose	Amperometry	NR (real-time)	0–0.7 mM	2.99 μM	850 μA·mM^−1^·cm^−2^	[84]
Sweat	PET	Carbon ink	Glucose	Amperometry	NR (real-time)	0.05 to 0.3 mM	NR	10.89 μA·mM^−1^·cm^−2^	[85]
	Tattoo	Carbon ink	Zinc	Amperometry	NR (real-time)	20–100 mM	0.05 μg/mL	23.8 μA mL/μg	[86]
	Carbon fibres	CNT ink	Na^+^	Potentiometry	NR	10^−6^ M to 10^−1^ M	4.02 × 10^−7^ M	0.19 mV/decade	[87]
	PET	GOx	Glucose	Amperometry	NR	NR	10 × 10^−6^ M	41.8 nA·µm^−1^·cm^−2^	[88]
	Polycarbonate	LOx	Lactate	Amperometry	NR	NR	NR	0.2 mM	[89]
	Tattoo	LOx	Lactate	Amperometry	NR (real-time)	1–20 mM	NR	0.1031 μA·mm^−2^·mM^−1^	[60]
		ETH 129, PANI	Ca^2+^, pH	Potentiometry	NR (real-time)	NR	NR	32.2 mV/decade, 62.5 mV/decade	[90]
	PDMS	GOx	Glucose	Amperometry	NR (real-time)	0–1 mM	NR	NR	[91]
Interstitial fluid	Tattoo	GOx	Glucose	Amperometry	NR	0–0.16 mM	NR	NR	[92]

NR: not reported; LOD: limit of detection; PET: polyethylene terephthalate; PB: polybutylene; PETG: polyethylene terephthalate Glycol; ETH 129: a thin organic membrane containing electrically neutral carrier calcium ionophore II; PDMS: polydimethylsiloxane; CNT: carbon nanotube; PANI: polyaniline; GOx: glucose oxidase; LOx: lactate oxidase.

**Table 3:** Comparison of representative printing approaches for electrochemical biosensors.

Method	Template Printing			Non-Template Printing	
	Screen Printing	Gravure Printing	Flexography Printing	Inkjet Printing	3D Printing
Ink viscosity (cP)	500–5000	100–1000	50–500	10–20	>300 k
Line width (μm)	50–100	10–100	45–100	2.3–50	1–100
Line thickness (μm)	3–250	1	<1	1–10	1–100
Speed (m/s)	70	1000	~500	~1	<1

**Table 4:** Summary of representative electrochemical sensors based on different substrates.

Substrate		Recognition Element	Analytes	Technique	Response Time	Limit of Detection	Flexible/Stretchable	Intimate Contact	Ref.
**Fabrics/Textiles**	Woven fiber	GOx	Glucose	FET	0.5 s	NR	Yes/Yes	No	[127]
	Textiles	PANI/PAN	Ammonia	Amperometry	9 s	10 ppm	Yes/Yes		[128]
		Carbon ink	TNT	Amperometry	NR	NR	Yes/NR		[118]
		Ionselective membranes	Na^+^, K^+^	potentiometry	NR (real-time)	10^−4.9^ M, 10^−4.9^ M	Yes/Yes		[129]
	Underwater garments	Tyrosinase	Phenols	Amperometry	NR	0.25 μM	Yes/NR		[130]
	Cotton	LOx	Lactate	Amperometry	NR	0.3 mM	Yes/NR		[131]
	Silk	LOx	Lactate	Amperometry	5 s	NR	Yes/NR		[132]
	Fabrics	GOx	Glucose	Amperometry	NR	NR	Yes/NR		[133]
Paper		GOx	Glucose	Amperometry	NR	NR	Yes/No	Yes	[134]
		Ag	Chloride	Amperometry	30–120 s	1.5 mM	Yes/No		[135]
		GOx	Glucose	Amperometry	NR	NR	Yes/No		[136]
		Ag	Chloride	Voltammetry	NR	NR	Yes/No		[137]
		Bienzymatic GOx-HRP	Glucose	Amperometry	NR	0.37 mg/dL	Yes/No		[138]
Plastic	Polyimide	Glutamate and LOx	Glutamate, lactate	Amperometry	A few seconds	220 nM, 2 mM	Yes/No	Yes	[139]
	Polyimide	LOx	Lactate	Amperometry	NR	NR	Yes/No		[140]
	PET	Enzymes	Uric acid, cholesterol, glucose	FET	NR	3 × 10^−9^ M, 30 × 10^−9^ M, 100 × 10^−9^ M	Yes/No		[141]
		LOx	Lactate	Amperometry	NR (real-time)	1.0 μM	Yes/No		[142]
	Polyamide	GOx	Glucose	Amperometry	NR	0.1 mg/dL	Yes/No		[143]
	PEN	LOx	Lactate	FET	NR (real-time)	66 nM	Yes/No		[144]
	Polyurethane	GOx	Glucose	Amperometry	NR (real-time)	0.010 mM	Yes/No		[145]
		LOx	Lactate	Amperometry	50 s	NR	Yes/No		[146]
Tattoo		Carbon ink	pH	Potentiometry	10 s	NR	Yes/No	Yes	[147]
		Ionselective membranes	Ammonium	Potentiometry	5 s	10^−4.9^ M	Yes/No		[148]
		Silver ink	Water content (hydration)	Impedimetry	NR	NR	Yes/No		[67]
		Alcohol oxidase	Ethanol	Potentiometry	30 s	NR	Yes/No		[149]

FET: field-effect transistor; PAN: polyacrylonitrile; HRP: horseradish peroxidase; NR: not reported.
